# Inhibition of Factor XI Using RBD4059: A Novel GalNAc-siRNA With Potent and Durable Antithrombotic Effects

**DOI:** 10.1016/j.jacbts.2024.12.005

**Published:** 2025-02-19

**Authors:** Junshi Liang, Sofia Nilsson, Johannes Wikström, Huiqing Cao, Shuquan Zheng, Qian Xu, Xu Yan, Sebastian Ueckert, Qi Sun, Chun Guo, Hongyan Zhang, Zicai Liang, Shan Gao, Li-Ming Gan

**Affiliations:** a Suzhou Ribo Life Science Co Ltd, Kunshan City, Jiangsu, China; b Ribocure Pharmaceuticals AB, Gothenburg, Sweden; c Department of Molecular and Clinical Medicine, Institute of Medicine, Sahlgrenska Academy, University of Gothenburg, Gothenburg, Sweden; d Department of Cardiology, Sahlgrenska University Hospital, Gothenburg, Sweden

**Keywords:** anticoagulation, factor XI, intrinsic pathway, siRNA, thrombosis

## Abstract

Coagulation factor XI (FXI) is an attractive target for novel antithrombotic drugs because of its minor role in hemostasis, which may address the current unmet medical need regarding a powerful anticoagulant with low bleeding risk. Here, we present RBD4059, the first FXI-targeting GalNAc-siRNA molecule to reach the clinical stage of development. In this study, RBD4059 demonstrated potent FXI inhibition with long duration in mice and monkeys, antithrombotic effects in mouse thrombosis models, and no prolonged bleeding in a mouse tail bleeding model. These findings make RBD4059 a promising candidate for the development of a safe and efficient antithrombotic drug.

## Highlights

• FXI is an attractive antithrombotic target because of its significant role in thrombosis and minor role in hemostasis.
• In the present study, the GalNAc-siRNA molecule RBD4059 showed promising reduction of FXI activity in preclinical animal models and antithrombotic effects without disrupting hemostasis.
• RBD4059 is the first FXI-targeting GalNAc-siRNA molecule to reach the clinical stage of development and is a promising candidate for the development of a safe and efficient antithrombotic drug with high patient compliance.

## Visual Abstract

**Figure:**
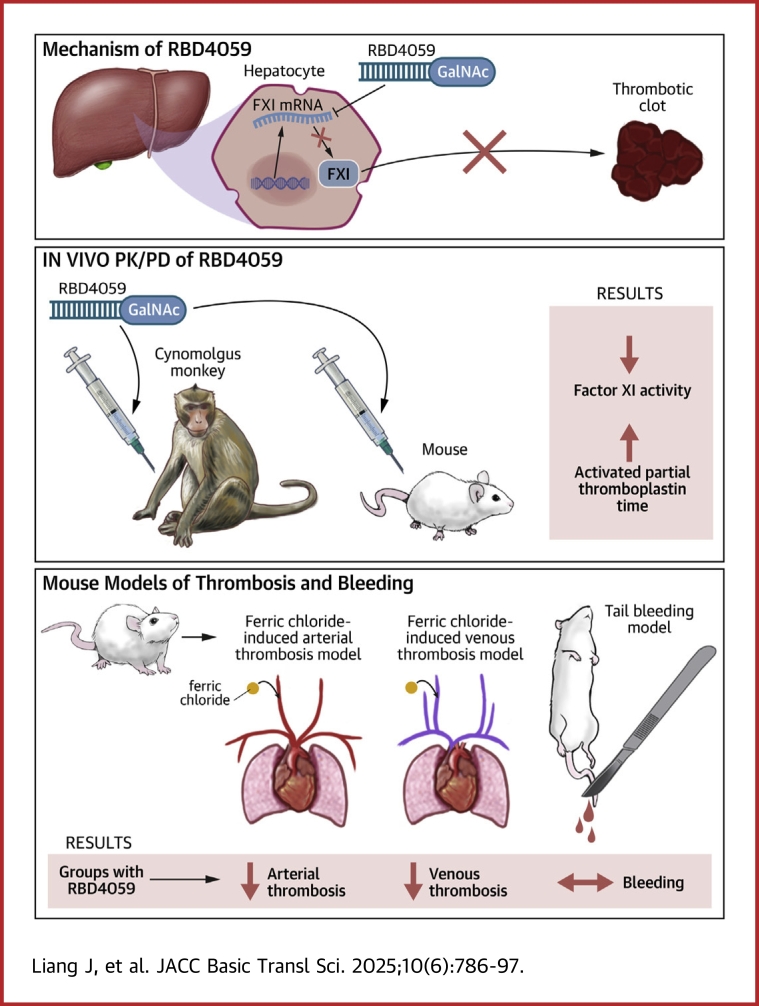

Thromboembolic diseases represent the leading cause of mortality and morbidity globally, and are estimated to account for 1 in 4 deaths worldwide.^1^ The mainstay for treatment and prevention of thromboembolic conditions is anticoagulation therapy.^2^ Currently existing anticoagulants, including vitamin K antagonists, unfractionated and low-molecular-weight heparins, and direct oral anticoagulants, can, however, be associated with serious bleeding complications as they target components of the extrinsic or common pathways of the coagulation cascade that are important for hemostasis and wound repair.^3^^,^^4^ Therefore, there is a major unmet medical need for developing novel antithrombotic strategies for safer treatment and prevention of thromboembolic diseases.

Components of the intrinsic pathway of coagulation have become attractive targets for the development of novel antithrombotic drugs, because their inhibition is less likely to cause bleeding complications.^5^ Factor XI (FXI) is the zymogen of the serine protease activated factor XI (FXIa) and is a major component of the intrinsic pathway of coagulation with a fundamental role in thrombosis.^6^^,^^7^ Previous studies have shown that patients with increased FXI levels have an increased risk of ischemic stroke and deep vein thrombosis.^8^^,^^9^ In contrast, inherited FXI deficiency (hemophilia C) may lead to a reduced risk of ischemic stroke and venous thromboembolism while it rarely manifests as spontaneous bleedings.^10^^,^^11^ Moreover, it has been demonstrated that inhibition of FXI with antibodies, antisense oligonucleotides, or small molecules has antithrombotic effects without increased risk of bleeding.^12^^,^^13^ These facts propose that components of the intrinsic coagulation pathway, particularly FXI, contribute to the pathogenesis in thromboembolic diseases, therefore making FXI a promising target for the development of safer anticoagulants.

Oligonucleotide-based therapies are an emerging class of human therapeutics with large clinical potential by silencing the production of disease-associated proteins at transcriptional level.^14^^,^^15^ Small interfering RNA (siRNA) therapy offers the advantage of selectively targeting the expression of nearly any mRNA of interest, and can thereby also inhibit targets that might be considered undruggable with conventional approaches.^16^ The precision of the siRNA typically also reduces risk of off-target effects after it is well-designed and chemically modified.^17^ In addition to molecular specificity, the possibility of targeting specific tissue or cells to achieve high efficacy delivery where it is needed has successfully been utilized in the development of both siRNAs and antisense oligonucleotides, thus also avoiding potential undesirable side effects and toxicity.^18^ Liver targeting represents a promising delivery strategy for antithrombotic drugs, because the liver is the major site for the synthesis of most blood coagulation factors.^19^ Conjugation of oligonucleotides to the liver targeted *N*-acetylgalactosamine (GalNAc) ligand facilitates receptor-mediated endocytosis by the asialoglycoprotein (ASGPR) receptor on hepatocytes.^20^ Therapeutic GalNAc-conjugated siRNAs have demonstrated long duration silencing in humans which enable less frequent administrations and compliance to treatment as compared to small molecules and therapeutic antibodies.^21^

In this study, we report the preclinical effects of RBD4059, the first GalNAc-siRNA molecule targeting FXI to reach clinic. By conjugating the FXI-targeting siRNA with the RIBO-GalSTAR delivery system to achieve a high degree of liver-targeting specificity, RBD4059 demonstrated efficient and durable down-regulation of FXI activity with prolonged activated partial thromboplastin time (APTT) and effects in thrombosis models. Based on preclinical data, RBD4059 is effective, durable, and well suited to be developed as a novel antithrombotic treatment with low bleeding risk. RBD4059 is currently investigated in a phase 1 clinical study in healthy human individuals (NCT05653037).

## Methods

### Animal experiments

The procedures related to animal testing comply with the relevant laws and regulations on the use and management of experimental animals and the relevant regulations of the institution’s IACUC (Institutional Animal Care and Use Committee, Laboratory Animal Care and Use Committee). Details of animal handling are described in the Supplemental Methods.

### Drug formulation

The RBD4059 siRNA drug was formed by hybridization of 2 chemically synthesized complementary RNA strands. The siRNA molecule was conjugated with a triantennary GalNAc group using the RIBO-GalSTAR liver targeting platform. The RBD4059 drug substance was manufactured by Asymchem Life Science (Tianjin) Co, Ltd in compliance with Good Manufacturing Process regulations.

### Bioinformatics analysis

The FXI transcripts of query species were acquired from the Gene Database of National Center for Biotechnology Information, and the siRNA sequence of RBD4059 was aligned for homology analysis by the DNAMAN software version 10 (Lynnon BioSoft).

### In vitro dual-luciferase reporter assay

The mouse FXI transcript and the FXI transcript shared between human and cynomolgus monkey containing the RBD4059 recognition site were inserted into the 3′ end of the *Renilla* reporter gene of the psiCHECK2 vector (Promega). HEK 293A cells were transfected using the Lipofectamine 2000 reagent (Invitrogen) according to the manufacturer’s protocol. The Dual-Glo Luciferase Assay System (Promega) was used for detection of the luminescence values of *Renilla* and *Firefly* luciferases in cell lysates 26 hours after transfection and treatment with RBD4059. Details of the dual-luciferase reporter assay are described in the Supplemental Methods.

### In vivo PK/PD studies in mouse

The concentration profile of the RBD4059 antisense strand in plasma was studied in CD-1 mice administered with a single subcutaneous dose of 1, 3, or 9 mg/kg RBD4059 (n = 9/sex/group). Plasma samples were collected at predose, and after 0.25, 0.5, 1, 2, 4, 6, 8, and 24 h. The antisense strand concentration was measured using liquid chromatography tandem mass spectrometry (LC-MS/MS) as described in the Supplemental Methods.

In a first pharmacodynamic (PD) study in C57BL/6J mice, 40 female mice were randomly divided into 5 groups (n = 8/group), including a phosphate buffered saline (PBS) (Macgene) control group and 4 RBD4059 groups administered with a single, subcutaneous dose of 1, 3, 6, or 9 mg/kg. Plasma FXI activity, APTT, and liver FXI mRNA levels were measured on day 8 after administration.

In a second pharmacokinetic (PK)/PD study in mice, female C57BL/6J mice were randomly allocated to 8 groups (n = 6/group), including 1 PBS control group and 7 groups administered with a single dose of RBD4059 at 9 mg/kg. APTT, FXI activity, and liver FXI mRNA levels were analyzed in the PBS group on day 1 after administration, and on day 2, 8, 15, 29, 43, 64, or 85 in the RBD4059 groups. At the same timepoints, liver samples were collected for RBD4059 antisense strand measurements using LC-MS/MS. Details of PK/PD methods and sample collection are described in the Supplemental Methods. Primer sequences used for RT-qPCR are listed in Supplemental Table 1.

### In vivo PK/PD studies in monkey

Cynomolgus monkeys were randomly divided into 3 groups (n = 3/sex/group) receiving a subcutaneous dose of 1, 3, or 9 mg/kg RBD4059. Plasma PK samples were collected at predose; after 10 and 30 min; and after 1, 2, 4, 6, 8, 10, 12, 24, 336, 672, 13,44, and 2,016 hours. Liver samples were collected on days 1, 2, 8, 15, 29, 43, 64, and 85 for the 9 mg/kg group. Measurement of the RBD4059 antisense chain concentration in plasma and liver samples was performed using LC-MS/MS. Blood collection for measuring FXI activity and APTT was performed at predose and on days 1, 2, 8, 15, 29, 43, 64, and 85.

### FeCl_3_-induced carotid artery and jugular vein thrombosis models

In the jugular vein thrombosis model, female C57BL/6J mice were randomly divided into 5 groups (n = 8/group): a PBS negative control group; RBD4059 1, 3, and 9 mg/kg groups; and an Enoxaparin sodium (4 mg/kg, Sanofi) positive control group. In the enoxaparin group, thrombosis modelling was induced 3 hours following administration. In the remaining groups, animals received a single subcutaneous dose followed by modelling of thrombus on day 8 after administration. After anesthesia, the jugular vein was dissected free and attached with a Doppler ultrasonic blood flow probe (Transonic) at the distal end. The blood flow velocity was measured using a TS420 perivascular flow module (Transonic) to confirm that the animal was in a stable condition by having a flow velocity of ≥0.2 mL/min for 2 minutes. Filter paper soaked in FeCl_3_ (5%, Macklin) was placed on the proximal side of the probe for 5 minutes to induce thrombosis. The blood flow velocity was continuously monitored and recorded for 25 minutes using the BIOPAC MP36 system, and data was extracted using the Biopac Student Lab 4.1 software.

In the carotid artery thrombosis model, male C57BL/6J mice were randomly divided into 5 groups (n = 10/group): a PBS (Solarbio) negative control group; RBD4059 1, 3, and 9 mg/kg groups; and an Enoxaparin sodium (4 mg/kg) positive control group. After anesthesia, filter paper soaked in FeCl_3_ (8%, Tianjin Fuchen) was used to induce local exposure to the proximal carotid artery for 5 minutes. The blood flow velocity was continuously monitored and recorded for 25 minutes using a Transonic T403 blood flow instrument, the BIOPAC MP160 system, and the AcqKnowledge software version 5.0.6.

### Mouse tail bleeding model

The mouse tail bleeding model was based on previously validated and described protocols.^22^^,^^23^ Briefly, female C57BL/6J mice were randomly divided into 3 groups (n = 10/group): PBS (Macgene) negative control group, RBD4059 (9 mg/kg) group, and Enoxaparin sodium (8 mg/kg) positive control group. RBD4059 was injected 1 week before the bleeding experiment, and enoxaparin was injected 4 hours before. After the mice were anesthetized, the tail of the mouse was removed at 1.5 cm from the tip of the tail using a disposable surgical blade, and the tail was immediately placed in a centrifuge tube filled with 4 mL 37 °C saline. The observation was continued for 30 minutes (1,800 seconds), and the time to first hemostasis and the total bleeding time were recorded.

### Statistical analysis

Data analysis and graph plotting were performed using R version 4.3.2.^24^ Data are represented as mean ± SEM unless otherwise specified. To assess statistical significance, outcome variables were log-transformed, and normality was evaluated using the Kolmogorov-Smirnov (KS) test. One-way analysis of variance, adjusted for baseline values when appropriate, was applied to test for significant differences between groups, followed by Tukey’s post hoc test for multiple comparisons. A *P* value <0.05 was considered statistically significant. Dose-response testing assuming a linear dose response relationship was performed using analysis of covariance using dose as continuous covariate. The results of the dose-response testing are shown in Supplemental Table 2.

## Results

### Inhibitory effects of RBD4059 on FXI in vitro

**Figure 1:**
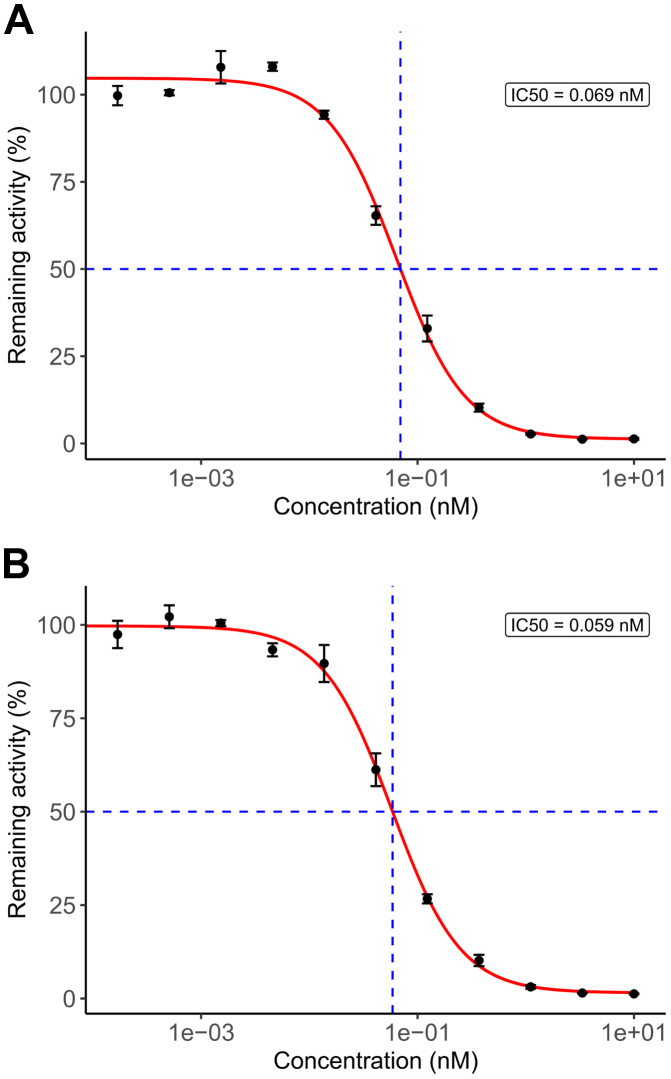
The In Vitro Inhibitory Activity of RBD4059 The inhibitory effect of RBD4059 on the factor XI transcript of (A) mouse and (B) human and monkey in a dual-luciferase reporter system. Data are shown as mean ± SEM. IC_50_ = half maximal inhibitory concentration.

A potent FXI-targeting siRNA was selected after addressing the in vitro potency of several siRNAs in the HepG2 cell line. Following conjugation of the candidate siRNA with GalNAc using the RIBO-GalSTAR liver targeting platform, the in vitro inhibitory activity of RBD4059 was evaluated on FXI transcripts of different species using a dual-luciferase reporter system. RBD4059 showed effective inhibition of the mouse FXI transcript with a half-maximal inhibitory concentration of 0.069 nmol/L, and an equivalent effect on the common FXI transcript for human and monkey (half-maximal inhibitory concentration of 0.059 nmol/L) (Figure 1). Homology analysis showed that RBD4059 is completely homologous to the FXI transcript of human, cynomolgus monkeys, Rhesus monkeys, and mice.

### Pharmacokinetic characteristics of RBD4059 in mouse and cynomolgus monkey

**Figure 2:**
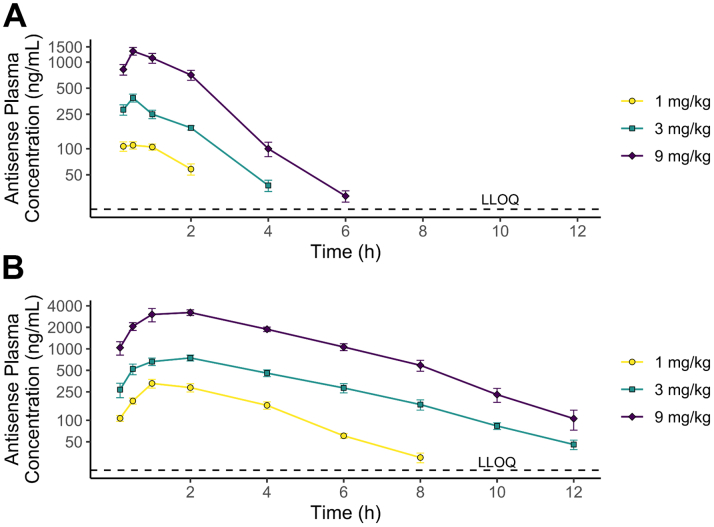
Pharmacokinetic Characteristics of RBD4059 in Mouse and Monkey Plasma concentration-time profiles of the RBD4059 antisense strand in (A) CD-1 mice (n = 18/group) and (B) cynomolgus monkey (n = 6/group) after a single subcutaneous dose at 1, 3, or 9 mg/kg. The LLOQ is 20 ng/mL. Data represents mean ± SEM. LLOQ = lower limit of quantification.

The PK characteristics of RBD4059 were evaluated in CD-1 mice and cynomolgus monkeys injected with a single subcutaneous dose of 1, 3, or 9 mg/kg. RBD4059 was rapidly absorbed into the blood of both mice and cynomolgus monkeys. In CD-1 mice, the peak plasma concentration was observed at 0.5 hours before it diminished promptly (Figure 2A, Supplemental Table 3). In the 3- and 9-mg/kg dose groups, the mean terminal elimination half-lives were 1.07 and 0.90 hours, respectively. In cynomolgus monkeys, RBD4059 showed a slightly prolonged half-life in blood as compared with the observations in mice. The peak plasma concentration was observed at 1.33, 1.67, and 1.67 hours in the 1-, 3-, and 9-mg/kg dose groups respectively, with the mean terminal elimination half-lives being 1.68, 1.97, and 1.55 hours (Figure 2B, Supplemental Table 4). The rapid removal from plasma indicates a rapid distribution of the drug to the targeted liver. A tissue distribution study in mice showed distinct liver uptake of the compound (Supplemental Figure 1).

### In vivo inhibitory effects of RBD4059 in mouse

**Figure 3:**
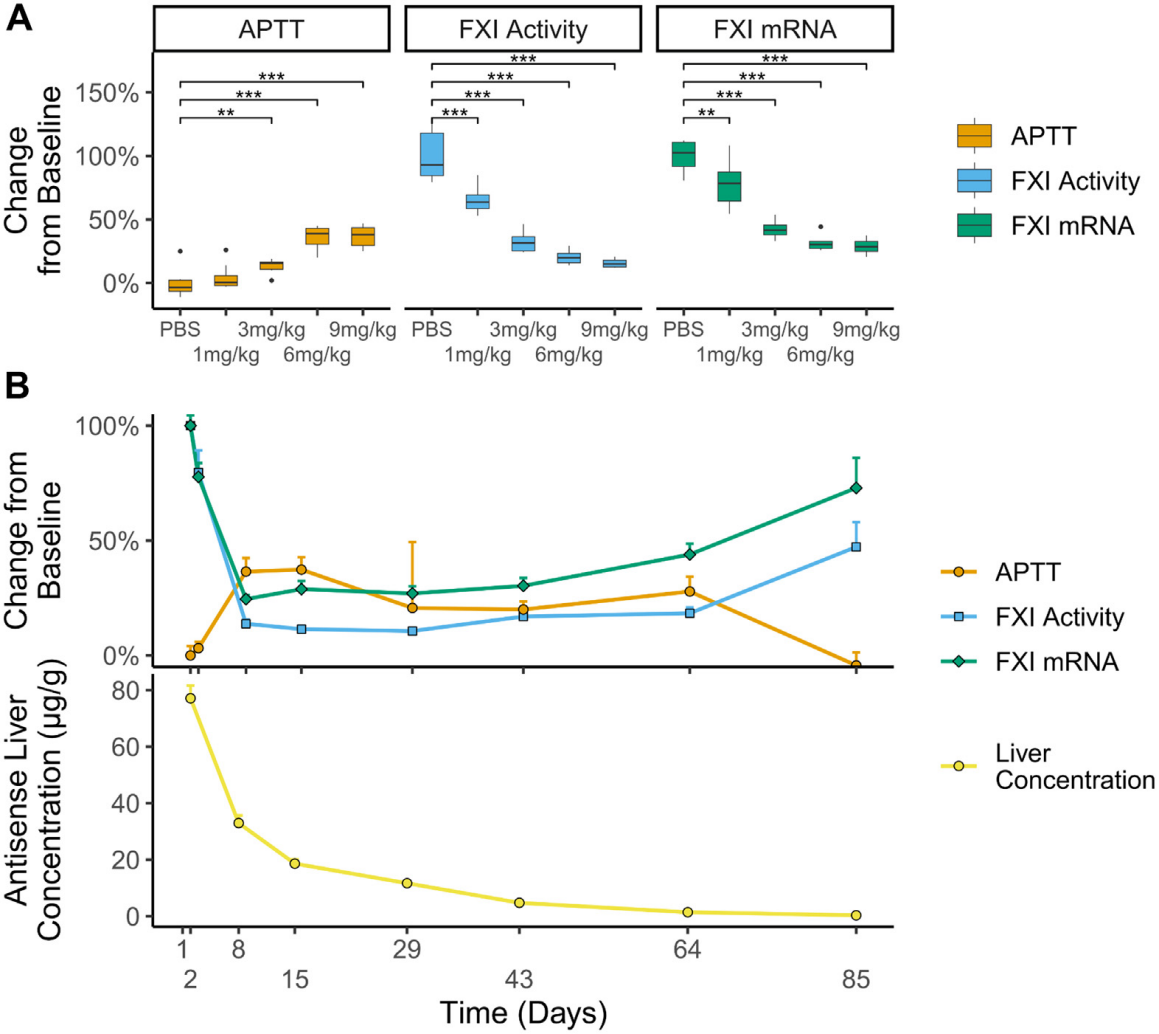
Pharmacodynamic Effects of RBD4059 in Mouse (A) Effect of a single dose of RBD4059 at 1, 3, 6, or 9 mg/kg on activated partial thromboplastin time (APTT), factor XI (FXI) activity, and liver FXI mRNA in mice 8 days following dosing (n = 8/group). (B) APTT, FXI activity, liver FXI mRNA level, and antisense liver concentration in mice after a single dose of RBD4059 at 9 mg/kg (n = 6/group). Data represents mean + SEM. Comparison of values across groups was performed using analysis of variance. ∗*P <* 0.05, ∗∗*P <* 0.01, ∗∗∗*P <* 0.001.

The in vivo inhibitory effect of RBD4059 was evaluated in C57BL/6J mice by administering a single subcutaneous dose of 1, 3, 6, or 9 mg/kg followed by PD analyses on day 8 after administration. RBD4059 exhibited significant effects on APTT prolongation, plasma FXI activity, and liver FXI mRNA levels, with the most pronounced effects observed with the highest dose (Figure 3A). Compared with PBS, the maximum APTT prolongation was approximately 1.4-fold *(P <* 0.001) after administration of 9 mg/kg RBD4059. The plasma FXI activity was reduced by 84.4% *(P <* 0.001), while the maximum inhibition of FXI mRNA reached 71.2% *(P <* 0.001). These results indicate that RBD4059 significantly increases APTT and reduces the activity of FXI by RNA interference-mediated degradation of hepatocyte FXI mRNA.

The durability of the inhibitory effects on PD parameters was evaluated by measuring at several time points in C57BL/6J mice treated with a single dose of RBD4059 at 9 mg/kg. The APTT data indicated a trend of APTT prolongation, with a maximum prolongation of approximately 1.4-fold on day 15. The maximum reduction of FXI activity level was 89.4% on day 29 after administration, and the maximum inhibition of FXI mRNA in the liver was 75.5% on day 8 after administration (Figure 3B). The PD parameters were observed to exhibit gradual recovery over time as the liver concentration of RBD4059 decreased, indicating that the inhibitory effects of RBD4059 are associated with its exposure in the liver.

### Long-lasting effects of RBD4059 in nonhuman primates

**Figure 4:**
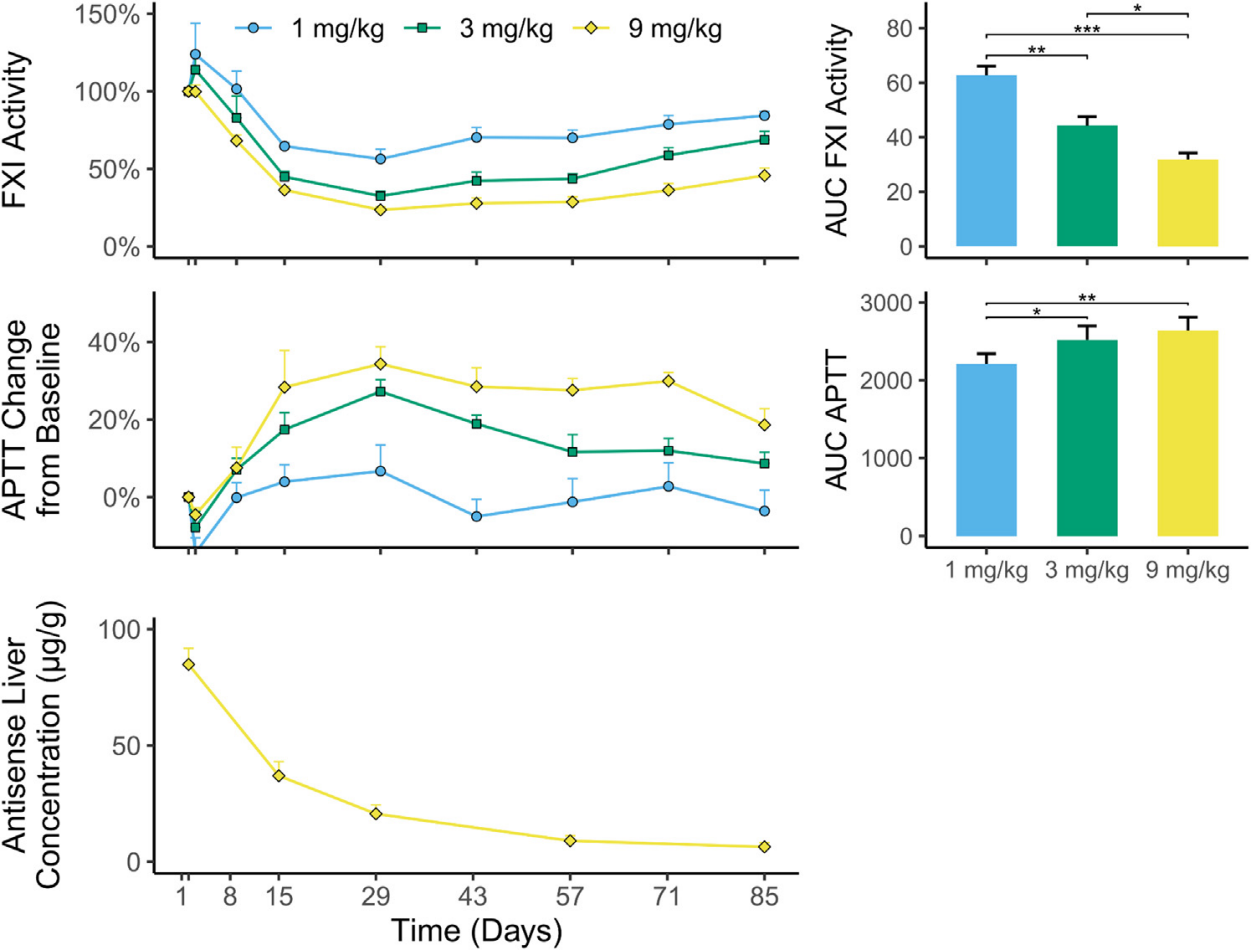
Pharmacodynamic Effects of RBD4059 in Monkey Effects of a single dose of RBD4059 at 1, 3, or 9 mg/kg on plasma FXI activity, APTT, and antisense liver concentration in cynomolgus monkeys (n = 6/group). (Due to the death of 1 test animal, the 9 mg/kg group has n = 5 from day 29.) Data represents mean + SEM. Comparison of AUC values across groups was performed using analysis of variance followed by Tukey's multiple comparison test. ∗*P <* 0.05, ∗∗*P <* 0.01, ∗∗∗*P <* 0.001. Abbreviations as in Figure 3.

The in vivo efficacy of RBD4059 was also evaluated in cynomolgus monkeys. In agreement with the findings in mice, RBD4059 exhibited long-lasting inhibition of FXI activity and prolongation of APTT in a dose-dependent manner in cynomolgus monkeys. A single dose of 9 mg/kg led to a maximum inhibition of FXI activity of 76.5%, with significant decrease in area under the curve (AUC) compared with the lower doses (Figure 4). Moreover, APTT was prolonged in monkeys administered with RBD4059, with a maximum prolongation of 1.34-fold on day 29 in the 9-mg/kg group. Treatment with RBD4059 resulted in significant increase in AUC for APTT for the 6- and 9-mg/kg groups as compared with the 1-mg/kg group. As observed in mice, the FXI activity and APTT recovered as the liver concentration of RBD4059 decreased slowly.

Taken together, these findings demonstrate that a single dose of RBD4059 is sufficient to provide long-lasting inhibition of FXI and prolongation of APTT, which is correlated with the decline of drug concentration in the liver. The slow elimination in the liver supports a low-frequency clinical administration of RBD4059.

### Antithrombotic effects of RBD4059 in arterial and venous thrombosis models

**Figure 5:**
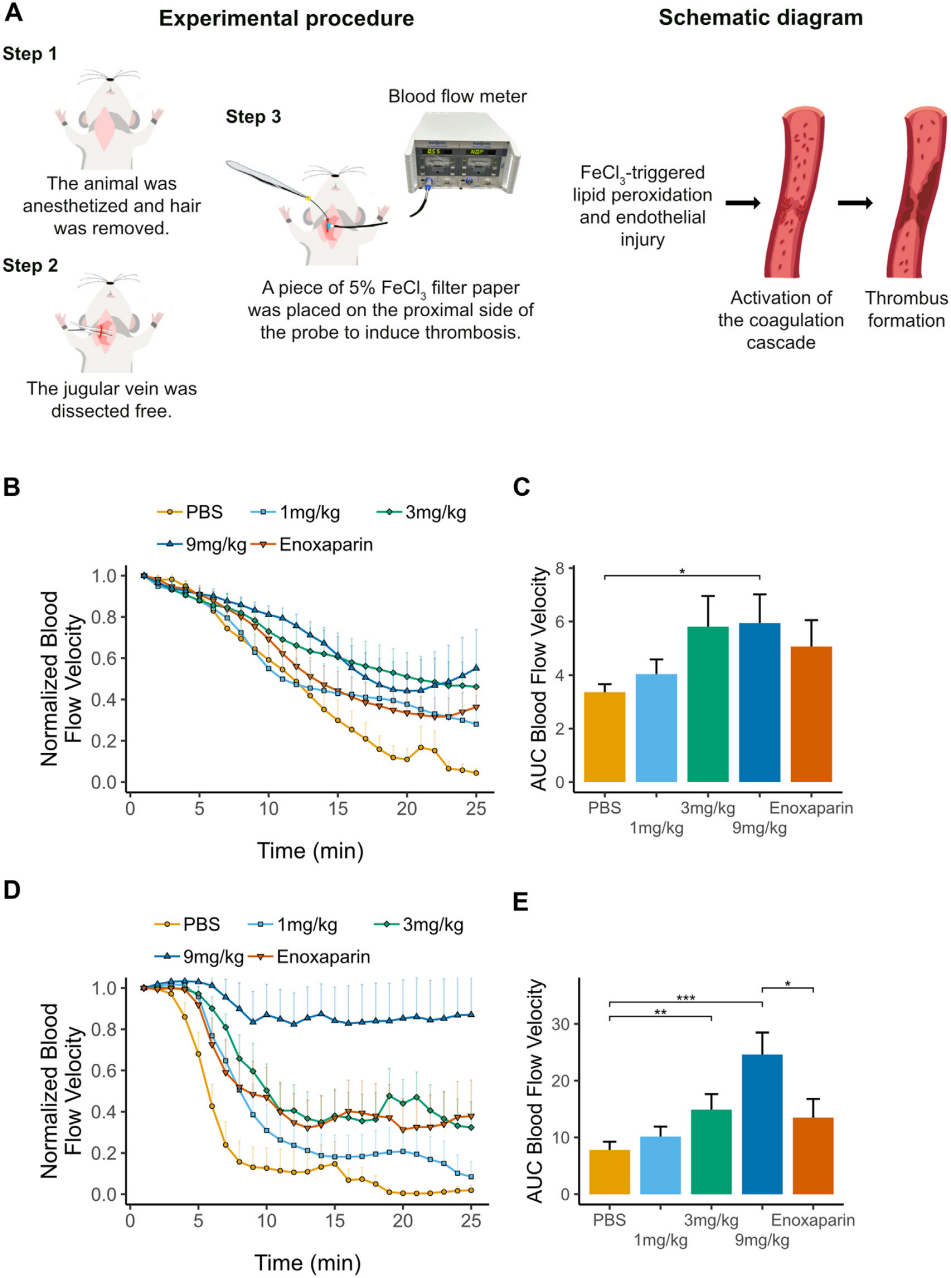
Antithrombotic Effects of RBD4059 in FeC3-Induced Thrombosis Mouse Models (A) Schematic representation of the ferric chloride (FeCl_3_)–induced jugular vein thrombosis mouse model. (B) Blood flow velocity for the jugular vein thrombosis model after a single subcutaneous dose of RBD4059 at 1, 3, or 9 mg/kg (n = 8/group). Enoxaparin at 4 mg/kg served as a positive control. Data represents mean + SEM. (C) AUC of the blood flow velocity in (B). (D) Blood flow velocity for the carotid artery thrombosis model after a single subcutaneous dose of RBD4059 at 1, 3, or 9 mg/kg (n = 10/group). Enoxaparin at 4 mg/kg served as a positive control. Data represents mean + SEM. (E) AUC for the blood flow velocity in (B). Comparison of values across groups was performed using analysis of variance. ∗*P <* 0.05, ∗∗*P <* 0.01, ∗∗∗*P <* 0.001. PBS = phosphate buffered saline.

The antithrombotic effect of RBD4059 was investigated in ferric chloride (FeCl_3_)-induced carotid artery and jugular vein thrombosis mouse models. In these models, FeCl_3_ is used to rapidly induce occlusive thrombosis in exposed veins or arteries during continuous monitoring of the blood flow velocity (Figure 5A). A subcutaneous injection of RBD4059 at 1, 3, or 9 mg/kg was administered 8 days before thrombosis modeling. In the jugular vein thrombosis model, RBD4059 prevented the reduction in blood flow velocity of the jugular vein, indicating effective prevention of thrombus formation and propagation that limits vessel occlusion (Figure 5B). RBD4059 at 9 mg/kg showed significant increase of AUC for blood flow velocity (*P <* 0.05) as compared with PBS (Figure 5C). The effect of RBD4059 was not significantly different to that of enoxaparin at 4 mg/kg, an equivalent dose used in clinic.

In the carotid artery thrombosis model, the effects of a single administration of RBD4059 on thrombosis inhibition lined up in a dose-dependent manner (Figure 5D). RBD4059 at 9 mg/kg prevented the decline in blood flow velocity caused by thrombosis, with a significant increase in AUC compared with PBS (*P <* 0.001) and enoxaparin (*P <* 0.05) (Figure 5E).

In summary, RBD4059 significantly prevented reduction in blood flow velocity in jugular vein and carotid artery thrombosis mouse models, indicating effective prevention of thrombus formation in vivo.

### Effect of RBD4059 on bleeding in the mouse tail bleeding model

**Figure 6:**
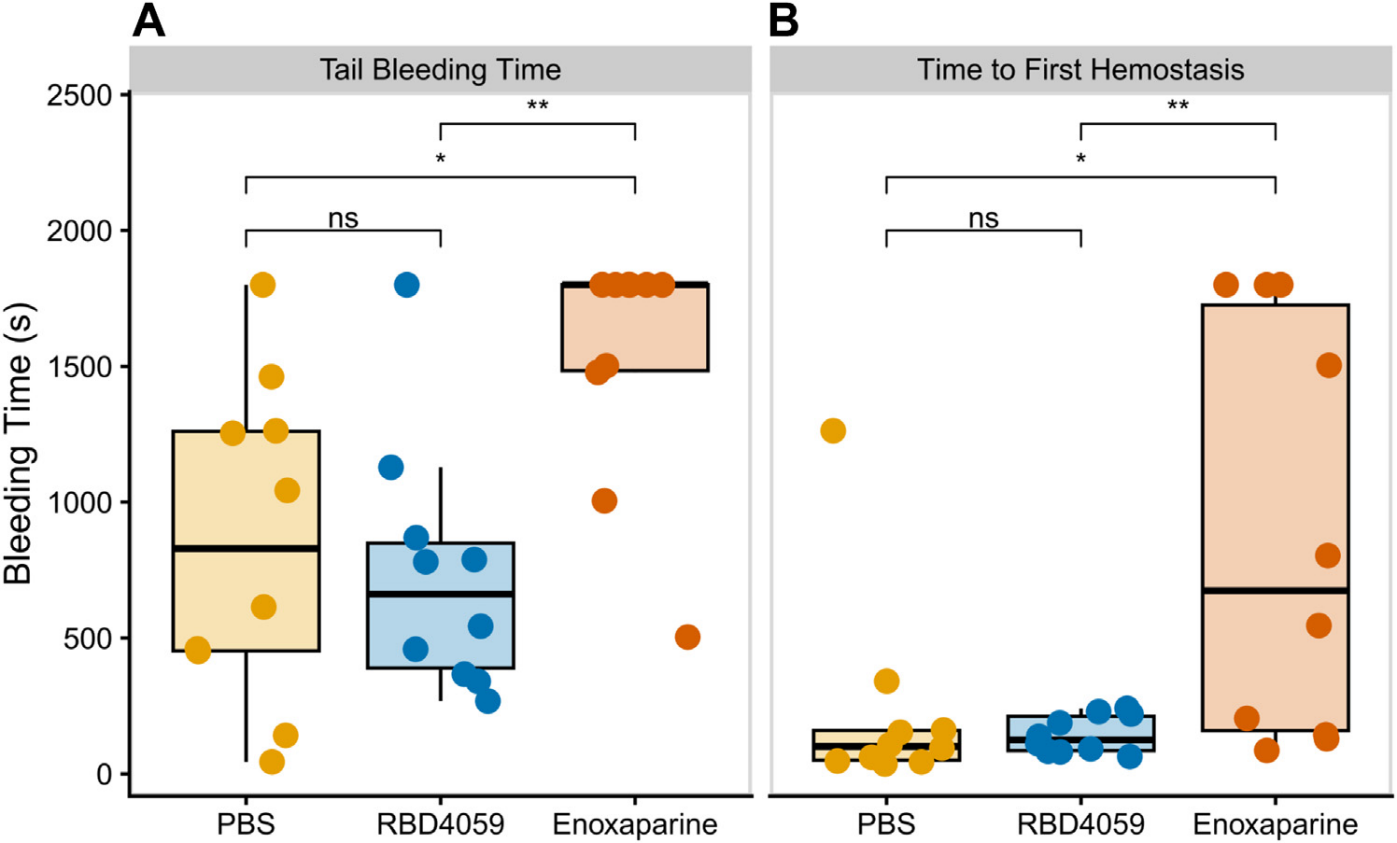
Effects of RBD4059 on Bleeding in a Mouse Tail Bleeding Model Effect of RBD4059 at 9 mg/kg on (A) total bleeding time and (B) time to first hemostasis in a mouse tail bleeding model (n = 10/group). Comparison of bleeding times across groups was performed using analysis of variance followed by Tukey's multiple comparison test. ∗*P <* 0.05, ∗∗*P <* 0.01. ns = not significant; PBS = phosphate buffered saline.

The effect of RBD4059 on hemostasis was assessed in a mouse tail bleeding model. After a single dose of RBD4059 at 9 mg/kg, the time to first hemostasis and the total bleeding time were not significantly different from those in the control group, whereas enoxaparin treatment led to significant increase in both parameters (*P <* 0.05) compared with the PBS group (Figure 6). These results indicate that treatment with RBD4059 is not impairing hemostasis in mice.

## Discussion

Currently existing anticoagulants can be associated with serious bleeding complications, which makes some patients ineligible for treatment.^4^ There is thus an unmet clinical need for the development of safer anticoagulants. Components of the intrinsic pathway of coagulation, especially FXI, have become attractive targets for the development of novel anticoagulants because they have minor roles in hemostasis and are thereby less likely to cause bleeding complications. Here, we present RBD4059, the first siRNA-based molecule targeting FXI to enter clinical development. In this study, RBD4059 demonstrated high potency and long duration regarding FXI inhibition in mice and cynomolgus monkeys. Notably, RBD4059 showed great antithrombotic potential because it efficiently prevented flow reduction in venous and arterial thrombosis mouse models.

siRNAs are an attractive class of therapeutics with certain advantages over other therapeutic approaches. Compared with traditional therapeutic approaches, GalNAc-siRNAs are less likely to be involved in drug-drug interactions including chemical inhibition or induction of drug-metabolizing enzymes and transporters.^25^ GalNAc-siRNA conjugates are also known to provide high potency and long duration of activity, which supports low-frequent drug administration and increased patient adherence.^21^^,^^26^ Moreover, the increased risk of bleeding from currently existing anticoagulants can be life-threatening for patients who exhibit uncontrolled bleeding or individuals who require surgical interventions. These therapeutic approaches therefore require the development of specific antidotes to reverse the risk of bleeding in emergency situations.^27^ By using siRNA to inhibit FXI at mRNA level, the effect of the drug can immediately be reversed at need using replacement therapy with fresh frozen plasma, which is an accepted method in clinical practice for the normalization of FXI levels in hemophilia C patients.^28^

Our siRNA approach targets FXI at the mRNA level, which subsequently leads to reduced levels of FXI, thus blocking the intrinsic pathway of coagulation. Another approach focuses on blocking the effects of FXIa. In line with other inhibitors of FXI and FXIa, inhibition of FXI using RBD4059 significantly increased the APTT in mice and cynomolgus monkeys in a dose-dependent fashion.^29^, ^30^, ^31^, ^32^ It has been indicated that APTT is strongly dependent on the level and activity of FXI.^33^^,^^34^ In patients with FXI deficiency, APTT typically becomes prolonged when the FXI level is reduced to <30%.^35^ This explains the more significant prolongation of APTT with the higher dose of RBD4059.

FXI inhibition using siRNA leads to lower FXI levels and thus replicates the clinical phenotype of congenital FXI deficiency. Patients with congenital FXI deficiency are known to have a reduced risk of venous thromboembolism and ischemic stroke and are not associated with a major risk of bleeding, which is consistent with the hypothesis that FXI has a minor role in hemostasis.^10^^,^^11^ Previous clinical studies of FXI and FXIa inhibitors provide supportive evidence that inhibition of FXI or FXIa is not associated with increased bleeding risk.^32^^,^^36^, ^37^, ^38^, ^39^, ^40^, ^41^ During the course of this study, no bleeding complications were observed in the test animals. The no observed adverse effect levels for RBD4059 were considered to be 600 mg/kg in mice and 400 mg/kg in monkeys in 12-week repeated dosing Good Laboratory Practice toxicity studies (unpublished data included in the IND documentation). Moreover, in contrast to enoxaparin, RBD4059 did not prolong the bleeding time nor hemostasis in a mouse tail bleeding model, which aligns with the assertion that FXI inhibition is safe in terms of bleeding risk.

A previous study demonstrated that FXI inhibition using an antibody prevented FeCl_3_-induced occlusion in the carotid artery to a comparable degree to total FXI deficiency.^42^ In the present study, RBD4059 demonstrated promising antithrombotic capacity as it significantly prevented reduction in blood flow velocity in both the carotid artery and jugular vein FeCl_3_-induced thrombosis mouse models, with superior efficacy compared with enoxaparin at the higher dose. This is in accordance with other FXI and FXIa inhibitors currently under clinical investigations that have demonstrated promising antithrombotic potential. Abelacimab, IONIS-FXI_RX_, and Milvexian have all demonstrated superior efficacy to enoxaparin in preventing postsurgical venous thromboembolism (VTE) in phase 2 trials.^36^, ^37^, ^38^

Another FXIa inhibitor that is currently under clinical investigation is the orally bioavailable small molecule Asundexian. In phase 1 evaluations, Asundexian showed dose-dependent inhibition of FXIa activity and increase in APTT without increased bleeding time.^31^^,^^43^ In a phase 2 study in patients with atrial fibrillation, Asundexian showed near-complete inhibition of FXIa at the highest dose.^39^ Despite the promising clinical potential of Asundexian observed in phase 1 and 2 trials, the OCEANIC-AF (A Study to Learn How Well the Study Treatment Asundexian Works and How Safe it is Compared to Apixaban to Prevent Stroke or Systemic Embolism in People With Irregular and Often Rapid Heartbeat [Atrial Fibrillation], and at Risk for Stroke) (NCT05643573) phase 3 trial was recently terminated early because of inferior efficacy in comparison to the activated factor X inhibitor Apixaban for prevention of stroke and systemic embolism in patients with atrial fibrillation.^44^ Although Abelacimab, IONIS-FXI_RX_, and Milvexian showed significant FXI activity inhibition-dependent clinical event reductions in their phase 2 postsurgical VTE trials, this piece of evidence is lacking for Asundexian. Indeed, Asundexian also used a proprietary self-developed FXI activity assay different from the commonly used clot-based FXI activity assay that was used in all the other trials mentioned above. Based on published phase 1 data from Asundexian, where also the widely used clot-based FXI activity assay was used in parallel and reported in the appendix (supporting information), the highest dose of 150 mg resulted in peak FXI inhibition around 50%.^43^ With the 50-mg dose selected in the OCEANIC-AF phase 3 trial, one may suspect that inferiority in efficacy may be caused by inadequate FXI inhibition. Despite the failure of Asundexian in its phase 3 trial, there is still strong belief in this target if meaningful degree of inhibition of FXI activity can be achieved. Based on all previously mentioned phase 2 trials in the postsurgical VTE setting, human genetic deficiency data, and preclinical thrombosis models, we believe that >80% inhibition of FXI antigen/activity, the latter measured in a clot-based FXI assay, will result in clinically meaningful efficacy in humans. Several large phase 3 trials are still ongoing, evaluating efficacy and safety of FXI inhibitors in high-risk patients who are currently not eligible for other antithrombotic therapies because of bleeding risks.^45^ Future studies will also show whether distinct indications may require different levels of FXI activity inhibition to balance risk and benefit profile in patients. With knowledge of the platform PK/PD characteristics of our siRNA molecules, RBD4059 is believed to be able to deliver a high degree of FXI inhibition (>95% upon repeated dosing according to internal unpublished modeling data), with 3 to 6 months duration in patients.

### Study limitations

The effects of RBD4059 in preclinical animal models might not fully reflect the effects of the drug in humans. It has been noted, eg, that GalNAc-siRNAs are more potent in humans than in mice and monkeys, with longer half-lives in the liver.^46^ It is therefore conceivable that the preclinical data may underestimate the true efficacy in humans. Based on a translational modeling approach, combining literature information and our in-house knowledge around species translation, we predict that we will achieve a high degree of FXI activity/antigen inhibition (>95%) via a different repeated dosing regimen. Further, the animal thrombosis models used are complex and to a large extent artificial, and they may not fully reflect the human atherothrombotic events. Thus, the optimal therapeutic dose required for clinical efficacy still needs to be explored in clinical trials.

## Conclusions

Based on the results obtained in this study, RBD4059 has proven to be a promising candidate for the development of a safe and effective anticoagulant with long durability. Further evaluation of RBD4059 is ongoing in a phase 1 study in healthy human individuals (NCT05653037).

Perspectives **COMPETENCY IN MEDICAL KNOWLEDGE:** Targeting components of the intrinsic pathway of the coagulation cascade, in particular FXI, can uncouple thrombosis from hemostasis to achieve antithrombotic effects without increased risk of bleeding. FXI inhibition based on an siRNA approach may provide distinct crucial clinical features, eg, high potency, long duration, and ready-to-go reversal strategies.

**TRANSLATIONAL OUTLOOK:** RBD4059 is proposed to be a safe and efficient antithrombotic drug that could benefit patients who are ineligible for currently existing treatment approaches because of bleeding risks. RBD4059 is currently being evaluated in a phase 1 study, which will lead to better understanding of its potential in humans.

## Funding Support and Author Disclosures

Drs Liang, Cao, Zheng, Xu, Yan, Sun, Guo, Zhang, Liang, Gao, and Gan are employees at Suzhou Ribo Life Science Co. Drs Nilsson, Wikström, Ueckert, and Gan are employees at Ribocure Pharmaceuticals AB.
