# Supplementary material for: Inhibition of Factor XI Using RBD4059: A Novel GalNAc-siRNA With Potent and Durable Antithrombotic Effects
Source: JACC Basic Transl Sci. 2025 Feb 19;10(6):786–97. doi: 10.1016/j.jacbts.2024.12.005 (PMC12230477; doi:10.1016/j.jacbts.2024.12.005)
Supplement: Supplemental Material [file mmc1.docx]

**Supplemental Appendix
List of Supplemental Materials**

Supplemental Methods2

Supplemental Figure 18

Supplemental Tables 1 to 49

**Supplemental Methods
Animal experiments**

The procedures related to animal testing in this study comply with the relevant laws and regulations on the use and management of experimental animals and the relevant regulations of the institution’s IACUC (Institutional Animal Care and Use Committee, Laboratory Animal Care and Use Committee). All animals were examined by a veterinarian before being assigned to the study.

C57BL/6J mice were supplied by SiPeiFu (Beijing) Biotechnology Co., Ltd. (laboratory animal production license number: SCXK (Beijing) 2019-0010). The mice were 6-8 weeks old when purchased and 7-9 weeks old when first administered. All animals were examined by a veterinarian upon receipt. Before the experiment, the animals were quarantined and adapted to the animal room for 5-8 days. The animals were kept in the Suzhou Ribo Animal Laboratory (Laboratory Animal Use License Number: SYXK (Beijing) 2019-0038).

PK experiments in CD1 mice were conducted at WuXi AppTec (Shanghai) Co. CD-1 mice (SPF) were supplied by Beijing Vital River Laboratory Animal Technology Co., Ltd. (laboratory animal production license: SCXK（京）2021-0006, laboratory male animal qualification number: 110011221107791982, laboratory female animal qualification number: 110011221107791651 and 110011221107791717). The age at dosing was 7-10 weeks. The rooms were controlled and monitored for relative humidity (targeted mean range 40-70%) and temperature (targeted mean range 20-26°C) with 10-20 air changes/hour. The room was on a 12-hour light/dark cycle except when interruptions were necessitated by study activities. The temperature and relative humidity were continuously monitored by Vaisala ViewLinc Monitoring system. Fresh drinking water and certified animal diet was available to all animals, ad libitum.

Animal experiments using cynomolgus monkeys were conducted at WuXi AppTec (Suzhou) Co., Ltd and WuXi AppTec (Shanghai) Co., Ltd. Naïve cynomolgus monkeys (≥ 2 years old) were supplied by Hainan Jingang Biotech Co., Ltd. (laboratory animal production license No.: SCXK（琼）2020-0001) and GuangDong Blooming-Spring Biological Technology Development Co., Ltd. (laboratory animal production license No.: SCXK（粤）2019-0027). Animals were acclimated for at least 14 days before being placed in the study. The rooms were controlled and monitored (WebCTRL system) for relative humidity targeted range 40-70%, and temperature targeted range 18-26°C with 10-20 air changes/hour. The rooms were on a 12-hour light/dark cycle except when interruptions were necessitated by study activities. Fresh drinking water was available to all animals, ad libitum. Animals were fed twice daily with approximately 120 grams of certified animal diet daily. In addition, animals received 100 grams of fruit daily as nutritional enrichment.

**Anesthesia prior to thrombosis and bleeding modelling**

Mice were anesthetized with 15 min of subcutaneous atropine (0.05 mg/ml; SC, 1 ml/kg), followed by intraperitoneal injection of a mixture of Zoletil® (50 mg/kg) and xylazine (12.5 mg/kg).

**Sample collection in mouse and monkey**

In order to collect plasma samples from mice, the animals were first anesthetized with a mixture of 70% CO_2_ and 30% O_2_. For PK analysis, approximately 0.1 ml blood was collected at each time point via saphenous vein puncture. Blood samples were transferred into tubes containing K_2_-EDTA. Plasma samples were then prepared by centrifugation at 3200 × *g* for 10 min at 4°C. Plasma samples were then quickly frozen over dry ice and kept at -60°C or lower until LC-MS/MS analysis. For PD analyses, blood was collected from the end of the orbital canthus venous plexus and 3.2% trisodium citrate dihydrate (whole blood-anticoagulant ratio 9:1) was added prior to centrifugation at 4000 rpm for 10 min at 4°C to separate the plasma for APTT and FXI activity detection.

Mouse liver tissue was collected after euthanasia. A gross dissection was performed, and the liver tissue was collected and cut into approximately 2 × 2 × 2 mm pieces. Liver samples for FXI mRNA detection were put into cryovials filled with RNAlater (Thermo Fisher Scientific) which were placed at 2-8°C for 24 hours prior to storage at -80°C. Tissue samples for RBD4059 liver concentration testing were placed in cryopreservation tubes, which were quickly frozen in liquid nitrogen prior to storage at -80°C.

For plasma collection in monkeys, 0.8 ml blood per animal and time point was collected from the peripheral veins. The blood was transferred to centrifuge tubes containing sodium citrate anticoagulant (whole blood-anticoagulant ratio 9:1), placed on wet ice, and centrifuged within 60 min at 1600 × *g* for 8 min at room temperature. Approximately 350 μl of plasma was placed on dry ice for quick freezing, and then stored at -60°C or lower until analysis. For liver tissue sample collection in cynomolgus monkeys, bright scan ultrasound was used to determine the location of the liver, generally focusing on the right liver. A biopsy gun was inserted into the liver and a puncture was taken. Samples greater than 7 mg were collected and homogenized by dilution 1:24 (w/v) in Clarity OTX Lysis-Loading buffer (Phenomenex) before being stored at -60°C or lower until PK analysis.

***In vitro* dual-luciferase reporter assay**

The FXI transcript of human and cynomolgus monkey (sense strand: 5’-GAGTACGTGGACTGGATTCTG-3’, anti-sense strand: 5’-CAGAATCCAGTCCACGTACTC-3’) and the mouse FXI transcript (sense strand: 5’AAGTACGTGGACTGGATTCTG-3’, anti-sense strand: 5’CAGAATCCAGTCCACGTACTT-3) containing the RBD4059 recognition site were inserted into the 3’ end of the *Renilla* reporter gene of the psiCHECK2 vector (Promega). The HEK 293A cell line was purchased from Nanjing Kebai Biotechnology Co., Ltd. One day prior to transfection, 8,000 cells were seeded in wells of a 96-well plate in DMEM (Gibco, Thermo Fisher Scientific) supplemented with 10% fetal bovine serum (Gibco, Thermo Fisher Scientific) at 37°C and 5% CO_2_. Transfection was performed using the Lipofectamine 2000 reagent (Invitrogen) according to the manufacturer’s protocol. Cells were treated with different concentrations of RBD4059 (0, 0.000169, 0.000508, 0.00152, 0.00457, 0.0137, 0.0412, 0.123, 0.370, 1.11, 3.33 and 10 nM). The Dual-Glo® Luciferase Assay System (Promega) was used for detection of the luminescence values of *Renilla* and *Firefly* luciferases in cell lysates 26 hours after transfection and treatment with RBD4059.

**APTT**

Plasma APTT was measured using a RAC-050 automatic coagulation analyser (Rayto) and the APTT reagent kit (Rayto) using the protocol: 50 μl of plasma sample, incubation 60 sec, 50 μl of APTT reagent, incubation 240 sec/120 sec, and 50 μl of CaCl_2_ solution.

**FXI activity assay**

FXI activity was measured in plasma samples using an APTT-based coagulation test on the RAC-050 automatic coagulation analyzer with the system: 10 μl of sample, 40 μl of diluent; 30 sec incubation, 50 μl of FXI-deficient plasma (Siemens); 30 sec of incubation, 50 μl of APTT reagent; 120 sec of incubation, and 50 μl of CaCl_2_ solution.

**LC-MS/MS**

Measurement of the RBD4059 antisense chain concentration in plasma and liver samples was performed by WuXi AppTec Co., Ltd. by using liquid chromatography tandem mass spectrometry (LC-MS/MS). The antisense strand of RBD4059 and the internal standard (PCSK9) were extracted by solid-phase extraction from liver homogenate or plasma. Reversed-phase ultra-performance liquid chromatography (UPLC) separation was achieved with a ACQUITY UPLC® BEH C18 300Å 2.1*100 mm I.D., 1.7 µm column (Waters). Mass spectrometry was performed using Triple Quad 6500^+^ (SCIEX), and MS/MS detections were set at mass transitions of m/z 844.0 → 94.9 and 628.7 → 94.9 for RBD4059 antisense strand and m/z 786.1 → 805.5 for IS in ESI negative mode. The LLOQ of RBD4059 was 20 ng/ml in plasma and 200 ng/g in liver homogenate.

**RNA extraction and RT-qPCR**

RNA was isolated from mouse liver tissue samples using 1 ml TRI Reagent (Sigma) and the protocol provided by the manufacturer. Reverse transcription was performed using the Reverse Transcription System (Promega). The reaction mixture was incubated at 42°C for 30 min, 95°C for 5 min, and 4°C for 5 min, and the cDNA product was diluted in 80 µl RNase-free water. RT-qPCR was performed using the following reaction mixture: 10 µl SYBR Select Master Mix (Applied biosystems), 0.5 µl forward primer (10 µM), 0.5 µl reverse primer (10 µM), 5 µl template, 4 µl RNase-free water. The reaction was incubated at 95°C for 5 min followed by 40 cycles of 95°C for 30 sec, 60°C for 30 sec, 72°C for 30 sec, and finally melt curve analysis at 60-95°C. GAPDH values were included for normalization. Primer sequences are listed in Supplemental Table 1.

**PK statistical analysis**

PK parameters were calculated by linear/log trapezoidal calculation method using Phoenix version 8.3.5 (Certara).

**Supplemental Figures and Legends**

**
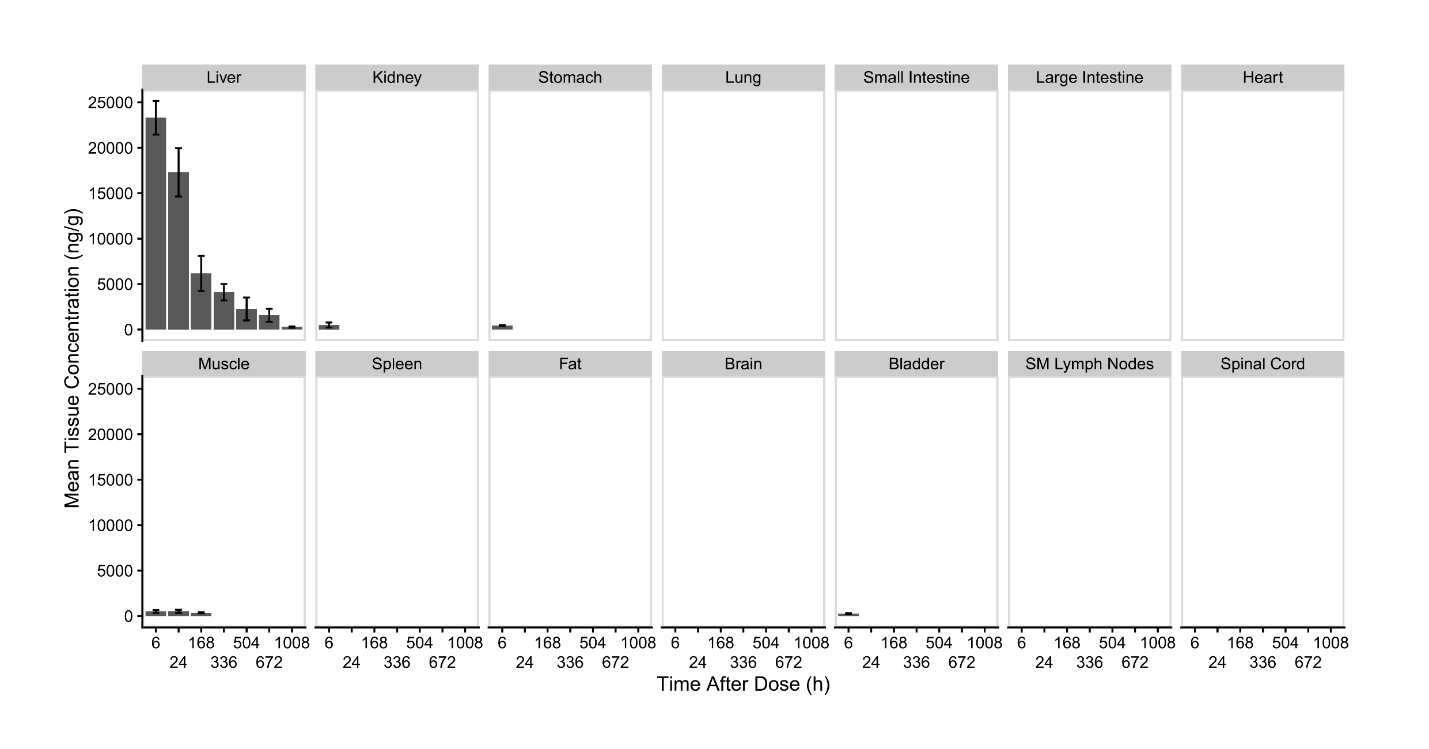
**

**Supplemental Figure 1. Tissue distribution of RBD4059 in mouse.** Tissue concentrations (ng/g) of RBD4059 antisense strand in CD-1 mice after a single subcutaneous administration of RBD4059 at 3 mg/kg (n = 3/sex/timepoint). Note: The concentrations in lung, small intestine, large intestine, heart, spleen, fat, brain, submaxillary lymph nodes and spinal cord were below the LLOQ (200 ng/g) at all study time points. Data represents mean ± SD. LLOQ, lower limit of quantification; SM, submaxillary.

**Supplemental Tables**

| **Primer name** | **Primer sequence 5’-3’** |
| --- | --- |
| Mouse FXI-F | GCCCTGTTAAAACTGGAATCAG |
| Mouse FXI-R | CGTTTCTATCTCCTTTGGAAGGC |
| Mouse GAPDH-F | TGCACCACCAACTGCTTAG |
| Mouse GAPDH-R | GGATGCAGGGATGATGTTC |

**Supplemental Table 1:** Primers used for hepatic mRNA expression analysis. F, forward; R, reverse.

| **Figure** | **Description** | ***P* value** |
| --- | --- | --- |
| 3 | APTT Dose Response | <0.001 |
| 3 | FXI Activity Dose Response | <0.001 |
| 3 | FXI mRNA Dose Response | <0.001 |
| 4 | AUC FXI Activity Dose Response | <0.001 |
| 4 | AUC APTT Dose Response | 0.0017 |
| 5 | Artery Blood Flow Dose Response | <0.001 |
| 5 | Vene Blood Flow Dose Response | 0.0573 |

**Supplemental Table 2:** Dose-response testing based on analysis of covariance (ANCOVA) using dose as a continuous covariate. APTT, activated partial thromboplastin time; AUC, area under the curve.

| **Dose level (mg/kg)** | **1** | **3** | **9** |
| --- | --- | --- | --- |
| **C_0_ or C_max_ (ng/mL)** | 110 | 387 | 1340 |
| **T_max_ (h)** | 0.5 | 0.5 | 0.5 |
| **t_1/2_ (h)** | NR | 1.07 | 0.901 |
| **AUC_0-last_ (h*ng/mL)** | 174 | 664 | 2620 |

**Supplemental Table 3:** PK parameters of RBD4059 antisense chain in CD1 mouse plasma after a single subcutaneous dose (*n* = 3 per sex per group). AUC_0-last_, area under curve from time zero to the last quantifiable concentration; C_0_, initial plasma concentration; C_max_, peak plasma concentration, NR, not reportable; T_max_, time at which C_max_ is achieved; t_1/2_, terminal elimination half-life.

| **Dose level (mg/kg)** | **1** | | **3** | | **9** | |
| --- | --- | --- | --- | --- | --- | --- |
|  | **Mean** | **SD** | **Mean** | **SD** | **Mean** | **SD** |
| **C_0_ or C_max_ (ng/mL)** | 345 | 117 | 759 | 172 | 3590 | 1090 |
| **T_max_ (h)** | 1.33 | 0.516 | 1.67 | 0.516 | 1.67 | 0.516 |
| **t_1/2_ (h)** | 1.68 | 0.357 | 1.97 | 0.594 | 1.55 | 0.357 |
| **AUC_0-last_ (h*ng/mL)** | 1200 | 230 | 3810 | 777 | 15900 | 2280 |

**Supplemental Table 4:** PK parameters of RBD4059 antisense chain in cynomolgus monkey plasma after a single subcutaneous dose (*n* = 3 per sex per group). AUC_0-last_, area under curve from time zero to the last quantifiable concentration; C_0_, initial plasma concentration; C_max_, peak plasma concentration; T_max_, time at which C_max_ is achieved; t_1/2_, terminal elimination half-life.
